# Age-stratified trends and outcomes of inpatient cholecystectomy for acute cholecystitis in the United States

**DOI:** 10.1016/j.sopen.2024.12.006

**Published:** 2024-12-28

**Authors:** Ayesha P. Ng, Joseph E. Hadaya, Sara Sakowitz, Zihan Gao, James Wu, Peyman Benharash

**Affiliations:** Department of Surgery, David Geffen School of Medicine at UCLA, Los Angeles, CA, USA

**Keywords:** Cholecystectomy, Gallstone disease, Age-stratified, Trends, Incidence, Outcomes

## Abstract

**Background:**

The elderly population in the United States is rapidly expanding. Older patients over age 65 with acute cholecystitis may face greater perioperative risk compared to younger patients undergoing urgent laparoscopic cholecystectomy. We aimed to characterize trends in utilization and outcomes of inpatient cholecystectomy across the United States stratified by age.

**Methods:**

All adults undergoing nonelective, laparoscopic cholecystectomy for acute cholecystitis in the 2012–2021 National Inpatient Sample were identified. Patients were stratified into 4 age groups: 18–49, 50–64, 65–79, and 80+ years. Major adverse events included in-hospital mortality and complications. Multivariable mixed regression was used to evaluate the association of age group with outcomes. Interaction terms were used to analyze differences in risk-adjusted outcomes over time.

**Results:**

Of 2,015,699 patients, 41.7 % were aged 18–49, 24.7 % were 50–64, 23.5 % were 65–79, and 10.2 % were 80+ years. Patients aged 65–79 and 80+ had major adverse event rates of 25 % and 34 %, respectively, compared to 5–14 % among younger patients (*p* < 0.001). After adjustment, patients over age 65 demonstrated nearly 2-fold greater odds of major adverse events (including repair of bile duct injury) and conversion to an open operation compared to younger patients. Patients aged 65–79 comprised an increasing proportion of cholecystectomy cases over time, from 20.0 % in 2012 to 27.5 % in 2021 (*p* < 0.001).

**Conclusions:**

Outcomes following cholecystectomy for acute cholecystitis among older patients remained significantly worse compared to younger patients over the past decade, with complication rates of 25–34 %. Preoperative counseling about the increased risk of complications following cholecystectomy for older patients is warranted.

## Introduction

Cholecystectomy is the most common intra-abdominal operation in the United States, with over 1.2 million cases performed annually [1]. In addition, the US population is rapidly aging, predominantly due to the baby boomer generation aged 65–79 that has significantly grown over the past decade [2]. As life expectancy continues to rise along with the epidemic of obesity, the prevalence of gallstone disease is increasing with age, and the elderly population is accounting for an escalating surgical burden [3]. A previous population-based study in 2011 suggested that patients >65 years of age have a >2-fold increased odds of mortality following inpatient cholecystectomy, relative to younger age groups, due to more complex biliary disease and lower physiologic reserve [4]. Nonetheless, improvements in surgical training and technology have improved the safety of laparoscopic cholecystectomy over time [5]. Decreasing rates of conversion to open cholecystectomy and greater knowledge of preoperative risk factors have contributed to a reduction in overall morbidity and costs [6]. Yet whether outcomes following cholecystectomy for elderly patients have improved with time remains unclear, as single-center studies suggest that postoperative morbidity remains significant despite careful patient selection [7].

Using a nationally representative cohort, the present study aimed to characterize age-stratified trends in utilization of nonelective, inpatient cholecystectomy for acute cholecystitis over the past decade. In addition, we examined the association of age with in-hospital clinical and financial outcomes. We hypothesized that older patients would demonstrate increasing rates of cholecystectomy over time and greater mortality, complications, and resource use compared to their younger counterparts.

## Methods

### Data source and study population

This was a retrospective cohort study using the 2012–2021 National Inpatient Sample (NIS). Maintained as part of the Healthcare Cost and Utilization Project, the NIS is the largest publicly available all-payer inpatient database in the United States [8]. Using survey weighting methodology, the NIS provides accurate estimates for approximately 97 % of all US hospitalizations. Due to the de-identified nature of the NIS, this study was deemed exempt from full review by the Institutional Review Board at the University of California, Los Angeles.

All adult patients (≥18 years) undergoing nonelective, laparoscopic cholecystectomy for acute cholecystitis were identified using relevant *International Classification of Diseases 9th/10th Revision* (ICD-9/10) diagnosis and procedure codes (Supplemental Table 1). Records with concomitant hepatic, biliary, pancreatic, or duodenal malignancies, chronic pancreatitis, pancreatic cysts, or liver transplantation were excluded from analysis. Patients were grouped into *18–49, 50–64, 65–79, and 80+* age strata.

### Demographic characteristics

Patient characteristics including age, sex, race, income quartile, and admission type were defined according to the NIS Data Dictionary [8]. The Elixhauser Comorbidity Index, a validated composite of 30 comorbidities, was used to quantify the overall burden of chronic conditions [9]. ICD-9/10 diagnosis codes were used to identify individual comorbidities and concomitant biliary diagnoses including choledocholithiasis, biliary colic, gallstone pancreatitis, and cholangitis (Supplemental Table 1). In accordance with the 2018 Tokyo Guidelines for acute cholecystitis, the most severe presentation (grade III) was defined as the presence of organ dysfunction using previous methodology [10]. Time to surgery was defined as the interval between admission and cholecystectomy. Procedures performed at the same hospitalization, including endoscopic retrograde cholangiopancreatography (ERCP), cholecystostomy tube placement, intraoperative cholangiogram, and common bile duct exploration, were identified using ICD-9/10 codes (Supplemental Table 1).

### Outcomes

Postoperative complications were classified into gastrointestinal (injury to the gastrointestinal tract, liver, or bile duct, retained stone), infectious (intraabdominal abscess, wound infection), respiratory (respiratory failure, prolonged mechanical ventilation, pneumonia), renal (acute kidney injury), cardiac (arrest, myocardial infarction), hemorrhagic, and thromboembolic (deep vein thrombosis, pulmonary embolism) (Supplemental Table 1). Conversion to open was identified using the appropriate ICD diagnosis code and any operation with open cholecystectomy procedure codes (Supplemental Table 1). Based on prior work, repair of bile duct injury was identified using ICD procedure codes for primary repair of bile ducts as well as surgical biliary reconstruction with hepaticojejunostomy or choledochojejunostomy following cholecystectomy [11]. Hospitalization costs were calculated via the application of center-specific cost-to-charge ratios to overall charges and inflation-adjusted using the 2021 Personal Healthcare Price Index [12]. The primary outcome of interest was major adverse events (MAE), a composite of mortality and postoperative complications. Secondary outcomes of interest included conversion to open, subtotal cholecystectomy, repair of bile duct injury, postoperative ERCP, length of stay (LOS), costs, and non-home discharge.

### Statistical analysis

Categorical and continuous variables are reported as frequencies (%) and medians with interquartile range (IQR) and were compared using the Pearson's chi-square and Mann-Whitney *U* tests, respectively. Multivariable mixed regression models were developed to evaluate the association of age group with outcomes of interest. Variable selection was performed by applying the Least Absolute Shrinkage and Selection Operator (LASSO) to enhance model generalizability and minimize overfitting and collinearity between independent variables [13]. Regression results are reported as adjusted odds ratios (AOR) for dichotomous and beta coefficients (β) for continuous variables with 95 % confidence intervals (95 % CI).

Significance of temporal trends was assessed using Cuzick's nonparametric test [14]. To determine the yearly incidence of cholecystectomy, the total US population was ascertained from the 2021 US Census data and used to calculate the number of cases per 100,000 US adults [15]. Incidence rate differences (IRD) were computed to compare annual cholecystectomy incidence over time [16]. Interaction terms between year of admission and age group were used to analyze risk-adjusted temporal trends in MAE. Statistical significance was set at α = 0.05. All statistical analyses were performed using Stata 16.1 (StataCorp, College Station, TX).

## Results

### Demographic comparison

Of an estimated 2,015,699 patients who underwent cholecystectomy, 840,030 (41.7 %) were aged 18–49 years, 497,175 (24.7 %) were 50–64 years, 473,870 (23.5 %) were 65–79 years, and 204,625 (10.2 %) were 80+ years. Compared to other age groups, the *80+* group had the greatest proportion of patients of White race and the highest income quartile (Table 1). In addition to acute cholecystitis, *80+* patients most frequently also presented with gallstone pancreatitis (*80+:* 16.9 % vs *65–79:* 16.6 % vs *50–64:* 15.4 % vs *18–49:* 13.5 %, *p* < 0.001), cholangitis (5.9 vs 3.9 vs 2.2 vs 1.1 %, p < 0.001), and Tokyo grade III disease (34.1 vs 25.0 vs 13.9 vs 4.8 %, *p* < 0.001). Furthermore, *80+* patients had the greatest time interval from admission to surgery (*80+:* 2 vs *65–79:* 1 vs *50–64:* 1 vs *18–49:* 1 days, *p* < 0.001) and most frequently underwent preoperative ERCP (18.1 vs 14.7 vs 11.6 vs 11.5 %, *p* < 0.001), cholecystostomy tube placement (0.4 vs 0.3 vs 0.2 vs 0.1 %, *p* < 0.001), intraoperative cholangiogram (24.1 vs 22.7 vs 22.0 vs 22.3 %, p < 0.001), and common bile duct exploration (1.1 vs 0.8 vs 0.5 vs 0.4 %, p < 0.001).

**Table 1:** Patient and treatment characteristics stratified by age group among patients undergoing cholecystectomy for acute cholecystitis. *IQR: Interquartile range.*

Parameter	18–49 years (*n* = 840,030)	50–64 years (*n* = 497,175)	65–79 years (*n* = 473,870)	80+ years (*n* = 204,625)	*p*-value
Female sex (%)	73.2	55.8	28.6	52.1	<0.001
Race (%)					<0.001
White	47.8	63.9	73.5	78.7	
Black	11.5	10.5	7.5	5.0	
Hispanic	32.7	18.0	12.3	10.4	
Asian	2.6	3.3	3.6	3.5	
Other	5.7	4.3	3.2	2.4	
Income Quartile (%)					<0.001
Fourth (highest)	17.5	21.7	21.5	22.7	
Third	23.9	25.0	25.2	25.6	
Second	26.3	25.4	26.7	26.7	
First (lowest)	32.3	27.9	26.5	25.0	
Comorbidities (%)					
Elixhauser Comorbidity Index (median, IQR)	1 [0–2]	2 [1–3]	3 [2–4]	3 [2–5]	<0.001
Diabetes	7.9	25.0	34.0	29.2	<0.001
Hypertension	17.8	55.2	75.1	81.9	<0.001
Coronary artery disease	1.1	9.5	22.6	29.9	<0.001
Congestive heart failure	1.1	5.4	12.2	20.9	<0.001
Chronic lung disease	8.9	13.3	17.3	17.6	<0.001
Chronic kidney disease	0.6	1.9	2.2	1.5	<0.001
Concomitant Diagnosis (%)					
Choledocholithiasis	0.4	0.4	0.4	0.5	<0.001
Biliary colic	0.5	0.5	0.6	0.6	<0.001
Gallstone pancreatitis	13.5	15.4	16.6	16.9	<0.001
Cholangitis	1.1	2.2	3.9	5.9	<0.001
Tokyo grade III disease (%)	4.8	13.9	25.0	34.1	<0.001
Treatment Characteristics (%)					
Preoperative ERCP (%)	11.5	11.6	14.7	18.1	<0.001
Cholecystostomy tube (%)	0.1	0.2	0.3	0.4	<0.001
Time to surgery (days, median, IQR)	1 [0–2]	1 [0–2]	1 [1–3]	2 [1–3]	<0.001
Intraoperative cholangiogram (%)	22.3	22.0	22.7	24.1	<0.001
Common bile duct exploration (%)	0.4	0.5	0.8	1.1	<0.001

### Postoperative outcomes

Compared to younger age groups, the *80+* group required the highest rates of conversion to open cholecystectomy (*80+:* 12.1 % vs *65–79:* 11.3 % vs *50–64:* 8.9 % vs *18–49:* 4.2 %, *p* < 0.001) and repair of bile duct injury (0.4 vs 0.3 vs 0.2 vs 0.2 %, *p* < 0.001, Table 2). The *65–79* and *80+* groups experienced higher rates of subtotal cholecystectomy (*80+:* 0.9 % vs *65–79:* 1.0 % vs *50–64:* 0.8 % vs *18–49:* 0.4 %, *p* < 0.001). Furthermore, *80+* patients demonstrated the greatest rates of MAE (*80+:* 34.4 %% vs *65–79:* 25.1 % vs *50–64:* 14.2 % vs *18–49:* 5.6 %, p < 0.001), including postoperative mortality (2.1 vs 1.0 vs 0.4 vs 0.1 %, p < 0.001) and complications, primarily renal, respiratory, gastrointestinal, and infectious (Table 2). Postoperative ERCP was most frequently performed among the *18–49* and *80+* cohorts (*80+:* 4.8 % vs *65–79:* 4.3 % vs *50–64:* 4.0 % vs *18–49:* 4.0 %, *p* < 0.001). Of note, the *80+* cohort experienced the greatest LOS (*80+:* 5 vs *65–79:* 4 vs *50–64:* 3 vs *18–49:* 2 days, p < 0.001), costs ($17,600 vs $15,400 vs $13,300 vs $11,300, p < 0.001), and rates of non-home discharge (31.5 vs 12.3 vs 4.6 vs 1.6 %, p < 0.001, Table 2).

**Table 2:** Unadjusted outcomes stratified by age group among patients undergoing cholecystectomy for acute cholecystitis. *IQR: Interquartile range. Major adverse events include mortality and any complication.*

Outcome	18–49 years (n = 840,030)	50–64 years (n = 497,175)	65–79 years (n = 473,870)	80+ years (n = 204,625)	p-value
In-hospital mortality (%)	0.1	0.4	1.0	2.1	<0.001
Complications (%)					
Gastrointestinal	1.5	2.3	3.2	3.9	<0.001
Infectious	0.4	1.0	1.9	2.7	<0.001
Respiratory	1.3	3.7	6.5	9.8	<0.001
Renal	2.2	8.2	16.5	23.5	<0.001
Cardiac	0.2	0.6	1.3	2.1	<0.001
Hemorrhagic	0.5	0.6	0.8	1.0	<0.001
Thromboembolic	0.1	0.3	0.5	0.7	<0.001
Major adverse event (%)	5.6	14.2	25.1	34.4	<0.001
Conversion to open (%)	4.2	8.9	11.3	12.1	<0.001
Subtotal cholecystectomy (%)	0.4	0.8	1.0	0.9	<0.001
Repair of bile duct injury (%)	0.2	0.2	0.3	0.4	<0.001
Postoperative ERCP (%)	4.8	4.0	4.3	4.8	<0.001
LOS (days, median, IQR)	2 [2–4]	3 [2–5]	4 [3–6]	5 [3–8]	<0.001
Costs ($1000s, median, IQR)	11.3 [8.5–15.4]	13.3 [9.8–18.7]	15.4 [11.1–22.0]	17.6 [12.8–25.2]	<0.001
Non-home discharge (%)	1.6	4.6	12.3	31.5	<0.001

**Fig. 1:**
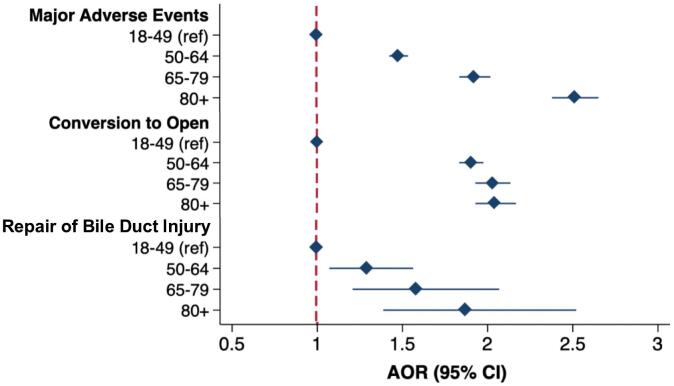
Adjusted outcomes associated with age group (reference: 18–49 years) among patients undergoing cholecystectomy for acute cholecystitis. *Ref: Reference. AOR: Adjusted odds ratio. CI: Confidence Interval. Major adverse events include mortality and any complication (gastrointestinal, infectious, respiratory, renal, hemorrhagic, and thromboembolic).*

After adjustment for the factors in Supplemental Table 2, the *80+* cohort remained associated with the greatest odds of MAE (AOR 2.51 [95 % CI 2.38–2.65]) relative to the 18–49 cohort (Fig. 1, Supplemental Table 3). In addition, the *80+* group demonstrated the greatest odds of conversion to open (AOR 2.03 [95 % CI 1.92–2.16]), repair of bile duct injury (AOR 1.88 [95 % CI 1.39–2.53]), and non-home discharge (AOR 8.73 [95 % CI 8.15–9.34]). Furthermore, *80+* age was associated with the greatest incremental increase in LOS (ß +1.5 days, 95 % CI 1.4–1.6) and hospitalization costs (ß + $2800, 95 % CI $2500-3100) compared to *18–49*.

### Trend analyses

**Fig. 2:**
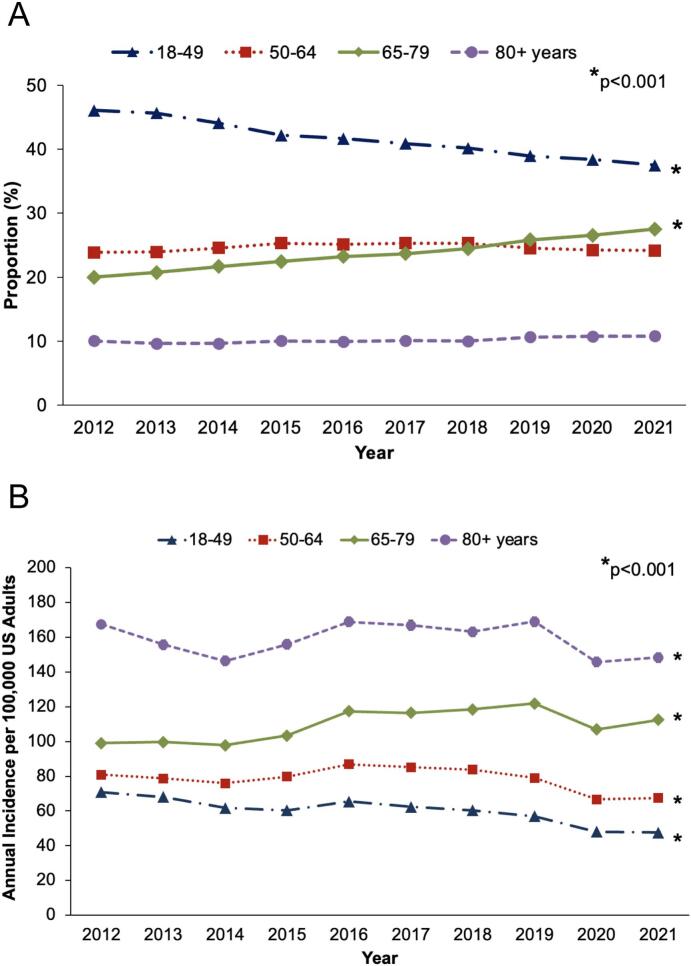
Trends in (A) proportion and (B) annual national incidence of nonelective, inpatient cholecystectomy for acute cholecystitis stratified by age group.

Over the 10-year study period, patients aged *65–79* comprised a significantly increasing proportion of inpatient cholecystectomy cases from 20.0 % in 2012 to 27.5 % in 2021 (*p* < 0.001), while operations among patients aged *18–49* decreased (Fig. 2A). Among the overall US population, the annual incidence of inpatient cholecystectomy increased by +13.4 per 100,000 adults among patients aged *65–79* (95 % CI 12.0–14.7) and decreased by −23.3 per 100,000 adults among patients aged *18–49* (95 % CI -22.7, −23.9, Fig. 2B).

**Fig. 3:**
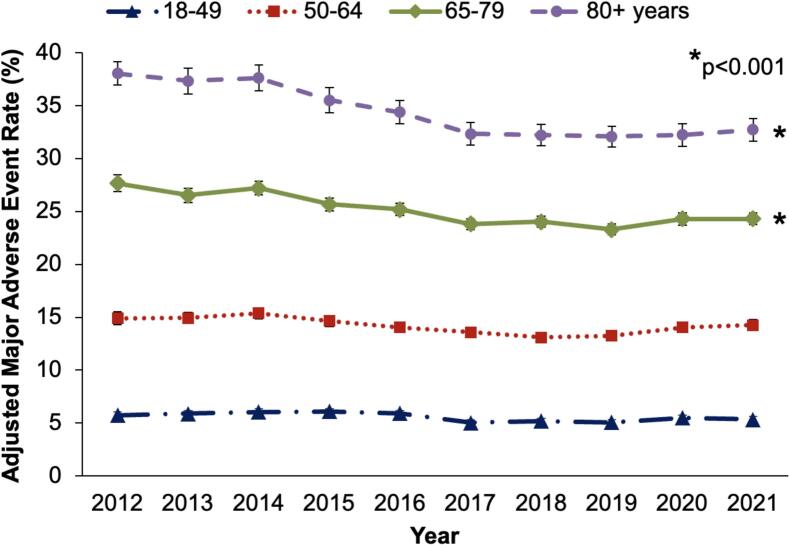
Trends in risk-adjusted rates of major adverse events by age group among patients undergoing cholecystectomy for acute cholecystitis. *Major adverse events include mortality and any complication (gastrointestinal, infectious, respiratory, renal, hemorrhagic, and thromboembolic).*

Following risk adjustment, MAE rates declined as an aggregate for the entire cohort from 16.4 % to 14.8 % over the study period (*p* < 0.001). When evaluated by age, MAE rates remained stable for patients under age 65 (Fig. 3). However, for patients aged *65–79*, a significant reduction in MAE from 27.7 % to 24.3 % was observed, as was a reduction from 38.0 % to 32.7 % for those aged *80+* (*p* < 0.001).

**Fig. 4:**
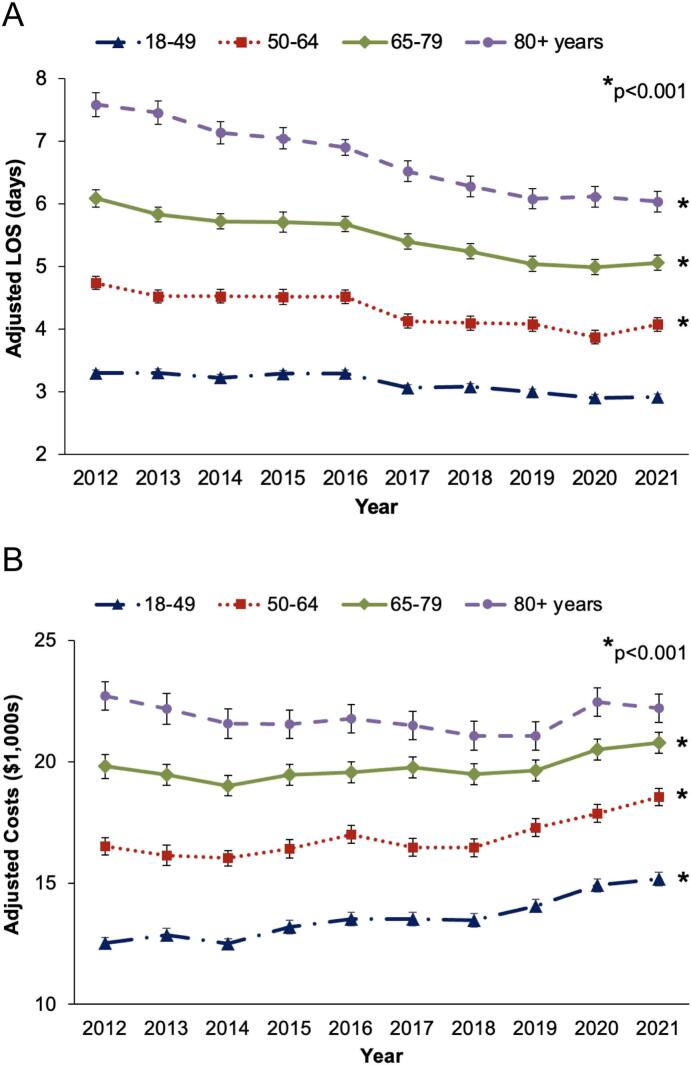
Trends in risk-adjusted (A) length of stay (LOS) and (B) hospitalization costs by age group among patients undergoing cholecystectomy for acute cholecystitis.

Over the 10-year study period, overall risk-adjusted LOS declined from 4.7 to 4.0 days (*p* < 0.001). When stratifying by age, LOS significantly decreased among patients aged 50 and older (*50–64*: 4.7 to 4.1 days, *65–79*: 6.1 to 5.1 days, *80+*: 7.6 to 6.0 days, Fig. 4A). Overall adjusted hospitalization costs increased from $16,300 to $18,000 over the study period (p < 0.001). On age-stratified analysis, costs significantly increased among all patients under age 80 (*18–49*: $12,500 to $15,200, *50–64*: $16,500 to $18,500, *65–79*: $19,800 to $20,800, p < 0.001, Fig. 4B).

## Discussion

Using a nationally representative cohort, the present study characterized trends and outcomes of inpatient cholecystectomy for acute cholecystitis stratified by age. Older age cohorts demonstrated significantly greater odds of major adverse events, conversion to open, and repair of bile duct injury compared to younger counterparts. Furthermore, over the 10-year study period, the average age of adults undergoing inpatient cholecystectomy significantly increased, with older adults comprising a larger proportion of patients undergoing cholecystectomy over time. Although rates of major adverse events have significantly reduced over the past decade, the overall rate remained markedly high among patients above age 65.

The higher complication rates we observed among older patients are consistent with existing literature. In a national 2011 study, nearly 40 % of patients above age 80 experienced complications, primarily infectious and respiratory, compared to 20 % among younger patients [4]. Mortality similarly increased with advancing age, from 0.4 % among patients aged 50–64 to 3.2 % among patients over 80 years (*p* < 0.001) [4]. In addition, elderly patients have previously demonstrated significantly higher rates of conversion to open cholecystectomy, ranging from 8 to 13 % [4,17,18]. Notably, a more recent study of the National Surgical Quality Improvement Program (NSQIP) between 2008 and 2020 examined approximately 430,000 patients and demonstrated that old age (>80 years) was associated with over 16-fold increased odds of mortality and 2-fold greater composite morbidity, namely respiratory and septic complications, compared to patients younger than 40 years old [19]. In light of our findings, it seems that advanced age continues to be associated with higher rates of perioperative morbidity after cholecystectomy, and patients should be counseled accordingly. Although the introduction of laparoscopic approach for cholecystectomy has led to the general perception as a relatively low-risk, routine procedure, our findings highlight that older patients warrant more careful consideration throughout perioperative risk stratification, counseling, and care coordination. Further interventions to tailor risk stratification models to older patients and optimize postoperative recovery pathways would be beneficial for this at-risk population.

Interestingly, growing literature suggests that cholecystectomy for cholecystitis may be safe in select settings for elderly patients. A 2015 randomized clinical trial reported that postoperative complication rates following cholecystectomy for Tokyo grades I and II acute cholecystitis were comparable between elderly and younger patients (14 vs 14 %, *p* = 0.72), suggesting that age should not be a limiting factor for surgical management of mild to moderate cholecystitis [20]. Furthermore, early timing of surgery may be another protective factor for older patients, as a recent systematic review found no significant difference in adverse events between elderly and younger patients who underwent early cholecystectomy on index admission [21]. In addition, a previous national study of patients above age 65 found that failure to perform cholecystectomy for acute cholecystitis on index admission was associated with increased morbidity, mortality, and costs [22]. Of note, the elderly patients who died or developed pulmonary and cardiac complications after early cholecystectomy all suffered from severe preexisting comorbidities or poor preoperative clinical condition, highlighting the importance of a thorough preoperative health assessment [21]. Advancements in the quality of minimally invasive technology, surgeon familiarity with the laparoscopic technique, and knowledge of preoperative risk factors have also reduced the need for conversion to open cholecystectomy and its associated adverse outcomes among elderly patients in recent years [6,23]. Further efforts to facilitate timely detection and early cholecystectomy for elderly patients when the disease is mild may help improve outcomes for this at-risk population.

The present study has several limitations due to its retrospective nature and use of administrative data. The NIS does not capture outpatient cholecystectomy procedures, and the inpatients in this study may have more comorbidities or severe biliary disease compared to the overall population undergoing cholecystectomy. In addition, emergent cholecystectomy cases performed within 24 h under medical observation may not require inpatient admission, further contributing to underestimation of incidence. Granular clinical information including laboratory values, gallbladder imaging, operative duration, and anatomic complexity of each procedure were unable to be assessed. The clinical and financial endpoints analyzed were limited to the duration of admission and preoperative data such as prior percutaneous cholecystostomy tube placement or long-term outcomes such as readmissions, postdischarge mortality, and reoperations were not available. Furthermore, ICD coding is influenced by provider and center practices among participating hospitals in the NIS, and the transition from ICD-9 to ICD-10 may introduce variations in coding. For example, ICD-10 diagnosis codes for bile duct injury were particularly sparse. Therefore, procedure codes for bile duct injury repair were used and may have overestimated rates of this complication, as biliary reconstruction may have been performed for other indications outside of bile duct injury. Nevertheless, rates of bile duct injury repair reported in the present study ranged from 0.2 to 0.4 % and were comparable to prior literature (0.2–0.7 %) [11]. Despite these limitations, we utilized the largest all-payer inpatient database and robust statistical methods to enhance the generalizability of our findings at the national level.

In conclusion, the present study used a nationally representative database to demonstrate that older patients experience greater morbidity and mortality following cholecystectomy for acute cholecystitis compared to younger patients. Over the past decade, the elderly have been comprising a greater proportion of cholecystectomy operations and still demonstrate persistently higher rates of adverse events. Given the rapidly aging population and rising clinical and financial burden of gallstone disease, further efforts are warranted to optimize perioperative management and improve outcomes among older patients.

## CRediT authorship contribution statement

**Ayesha P. Ng:** Writing – review & editing, Writing – original draft, Validation, Methodology, Investigation, Formal analysis, Data curation, Conceptualization. **Joseph E. Hadaya:** Writing – review & editing, Methodology, Investigation, Data curation, Conceptualization. **Sara Sakowitz:** Writing – review & editing, Methodology, Data curation, Conceptualization. **Zihan Gao:** Writing – review & editing, Writing – original draft. **James Wu:** Writing – review & editing, Writing – original draft, Validation, Investigation, Conceptualization. **Peyman Benharash:** Writing – review & editing, Validation, Supervision, Software, Resources, Project administration, Methodology, Investigation, Conceptualization.

## Ethics approval

This study was deemed exempt from full review by the Institutional Review Board at the University of California, Los Angeles.

## Funding sources

The present work did not receive any funding.

## Declaration of competing interest

The authors declare no conflicts of interest.

## Appendix A Supplementary data

Supplementary tables Image 1
