# Supplementary material for: Age-stratified trends and outcomes of inpatient cholecystectomy for acute cholecystitis in the United States
Source: Surg Open Sci. 2024 Dec 28;23:24–9. doi: 10.1016/j.sopen.2024.12.006 (PMC11745976; doi:10.1016/j.sopen.2024.12.006)
Supplement: Supplementary file 1 — Supplementary tables [file mmc1.docx]

Supplemental Table 1. Administrative *International Classification of Diseases, 9^th^ and 10^th^ Revision* (ICD-9/10) diagnosis and procedure codes for cholecystectomy for acute cholecystitis.

|  | **ICD-9** | **ICD-10** |
| --- | --- | --- |
| **Cholecystitis** | 574.0x, 574.1x, 575.0, 575.1 | K80.0x, K80.1x |
| **Cholecystectomy** |  |  |
| Laparoscopic | 51.23, 51.24 | 0FT44ZZ, 0FB44ZZ, 0FB48ZZ, 0F544ZZ, 0F548ZZ |
| **Exclusions** |  |  |
| Hepatic, biliary, pancreatic, and duodenal malignancy | 152, 155, 156, 157, 158, 159 | C22, C23, C24, C25 |
| Chronic pancreatitis | 577.1 | K86.0, K86.1 |
| Pancreatic cyst | 577.2 | K86.2, K86.3 |
| Non-stone gallbladder disease | 576.2, 575.2-9 | K82.4, K82.8, K83.01, K83.1 |
| Liver transplant (procedure) | 50.51, 50.59 | 0FY00Z0, 0FY00Z1, 0FY00Z2 |
| Liver transplant (diagnosis) | 996.82 | T86.4 |
| **Tokyo grade III** |  |  |
| Hypotension | 458 | I95 |
| Acute respiratory failure | 518.8 | J96 |
| Altered mental status | 780.0 | R40 |
| Renal failure | 584, 586 | N17, N19 |
| Acute hepatic dysfunction | 570 | K72.0 |
| Thrombocytopenia | 287.4, 287.5 | D96.5, D69.6 |
| **Concomitant Gallstone Diagnosis** |  |  |
| Biliary colic | 574.2x | K80.2x, K80.8x |
| Choledocholithiasis | 574.5x, 574.9 | K80.5x, K80.7x |
| Cholangitis | 576.1 | K80.3x, K83.0x |
| Gallstone pancreatitis | 577.0 | K85.0-1x, K85.5x, K85.9x |
| **Comorbidities** |  |  |
| Diabetes | 250 | E10, E11, E13 |
| Hypertension | 401, 402, 403, 404, 405 | I10, I11, I12, I13, I15 |
| Coronary artery disease | 411, 412, 413, 414 | I20, I24, I25 |
| Congestive heart failure | 398.91, 402.01, 402.91, 404.01, 404.03, 404.11, 404.13, 404.91, 404.93, 425.4, 425.5, 425.7, 425.8, 425.9, 428 | I43, I50, I09.9, I11.0, I13.0, I25.5, I42.0, I42.5, I42.6, I42.7, I42.8, I42.9, P29.0 |
| Chronic lung disease | 416.8, 416.9, 506.4, 508.8, 500, 501, 502, 503, 504, 505, 490, 491, 492, 493, 494, 495, 496 | I27.8, I27.9, J68.4, J70.1, J70.3, J40, J41, J42, J43, J44, J45, J46, J47, J60, J61, J62, J63, J64, J65, J66, J67 |
| Chronic kidney disease | 403.01, 403.11, 403.91, 404.02, 404.03, 404.12, 404.13, 404.92, 404.93, 588.0, 585.5, 585.6, 586.5, 586.6 | Z49, N19, I12.0, N25.0, Z94.0, N18.5, N18.6, I13.11, V45.1, Z99.2, Z91.15 |
|  |  |  |
| **Treatment Characteristics** |  |  |
| ERCP | 51.10, 51.11, 51.8, 52.94, 52.97 | 0FJB8ZZ, 0FJD8ZZ, 0FB58ZX, 0FB68ZX, 0FB78ZX, 0FB88ZX, 0FB98ZX, 0FBC8ZZ, 0F758ZZ, 0F768ZZ, 0F778ZZ, 0F788ZZ, 0F798ZZ, 0F7C8ZZ, 0F9C8ZZ, 0F758DZ, 0F768DZ, 0F778DZ, 0F788DZ, 0F798DZ, 0F7C8DZ, 0F9580Z, 0F9680Z, 0F9780Z, 0F9880Z, 0F9980Z, 0F9C80Z, 0FC58ZZ, 0FC68ZZ, 0FC78ZZ, 0FC88ZZ, 0FC98ZZ, 0FCC8ZZ, 0FF48ZZ, 0FF58ZZ, 0FF68ZZ, 0FF78ZZ, 0FF88ZZ, 0FF98ZZ, 0FFC8ZZ, 0FCD8ZZ, 0FCF8ZZ, 0FFD8ZZ, 0FFF8ZZ, 0F9D80Z, OF9F80Z |
| Percutaneous cholecystostomy tube | 51.01 | 0F943ZZ |
| Intraoperative cholangiogram | 87.53 | BF030ZZ, BF031ZZ, BF03YZZ, BF0C0CZZ, BF0C1ZZ, BF0CYZZ, BF100ZZ, BF10YZZ, BF110ZZ, BF111ZZ, BF11YZZ, BF120ZZ, BF121ZZ, BF12YZZ, BF130ZZ, BF131ZZ, BF13YZZ, BF140ZZ, BF141ZZ, BF14YZZ, BF180ZZ, BF181ZZ, BF18YZZ |
| Common bile duct exploration | 51.41, 51.42, 51.49, 51.51 | 0FC90ZZ, 0FC50ZZ, 0FC60ZZ, 0FC70ZZ, 0FC80ZZ, 0FF53ZZ, 0FF57ZZ, 0FF60ZZ, 0FF63ZZ, 0FF67ZZ, 0FF70ZZ, 0FF73ZZ, 0FF77ZZ, 0FF80ZZ, 0FF83ZZ, 0FF87ZZ, 0FF90ZZ, 0FF93ZZ, 0FF97ZZ, 0FFC0ZZ, 0FFC3ZZ, 0FFC7ZZ |
| **Postoperative Outcomes** |  |  |
| Conversion to open | V64.41, 51.21, 51.22 | Z53.31, 0F540ZZ, 0F543ZZ, 0FB40ZZ, 0FB43ZZ, 0FT40ZZ |
| Subtotal cholecystectomy | 51.21, 51.24 | 0FB40ZZ, 0FB44ZZ |
| Repair of bile duct injury | 51.36-9, 51.71-9 | 0F15-9, 0FQ5-9, 0FR5-9, 0FU5-9 |
|  |  |  |
| **Complications** |  |  |
| Injury to the GI tract, liver, or bile duct | 863, 864, 868 | S36 |
| Retained stone | 997.41 | K91.86 |
| Bile duct fistula | 57.64 | K83.3 |
| Sphincter dysfunction | 57.65 | K83.4 |
| Postcholecystectomy syndrome | 57.60 | K91.5 |
| Other postoperative gastrointestinal complications | 997.49 | K91.3, K91.81, K91.82, K91.83, K91.89 |
| Sepsis | 038, 995.91, 995.92, 999.3, 998.51, 998.59 | A40, A41, R65.20, T814XXA, K68.11 |
| Abscess | 569.5, 790.7 | R78.81, K63.0 |
| Wound infection | 998.31, 998.32, 998.5 | T81.32XA, T81.31XA, T81.4XXA, K68.11 |
| Pneumonia | 480, 481, 482, 483, 485, 486, 997.31, 997.32 | J12, J13, J14, J15, J16, J18, J95.851, J95.89 |
| Pneumothorax | 512.1 | J95.811 |
| Acute respiratory distress syndrome | 518.5, 518.82 | J80, R06.03 |
| Respiratory Failure | 518.81, 518.51, 518.53, 518.84 | J96.00, J96.90, J96.20, J95.821, J95.822 |
| Prolonged Mechanical Ventilation | 96.72 | 5A1955Z |
| Deep Vein Thrombosis | 451.1, 451.2, 451.81, 451.9, 453.2, 453.40, 453.41, 453.42, 453.8, 453.9 | I82.220, I82.4, I82.6, I82.A1, I82.B1, I82.C1, I82.290, I82.890, I82.91, I80.9, I80.3 |
| Pulmonary Embolism | 415.1 | I26 |
| Cardiac arrest | 427.5 | I46.2, I46.8, I46.9 |
| Ventricular tachycardia | 427.1 | I47.2 |
| Ventricular fibrillation | 427.41 | I49.01 |
| Cardiac tamponade | 423.3 | I31.4 |
| Myocardial infarction | 410 | I21 |
| Stroke | 433.01, 433.11, 433.21, 433.31, 433.81, 433.91, 434.01, 434.11, 434.91, 437.0, 437.1, 437.4, 437.5, 437.7, 437.9, 997.01, 997.02, 431, 432.0, 432.1, 432.9, 430 | I63, I67.2, I67.81, I67.82, I67.89, I67.7, I67.5, I67.9, G97.81, G97.82, I97.811, I97.821, I61.9, I62.1, I60.9, I62.9, I62.0, I62.1 |
| Acute kidney injury | 584 | N17 |
| Hemorrhage | 998.11 | D78.01, D78.02, D78.21, D78.22, E36.01, E36.02, E89.810, E89.811, G97.31, G97.32, G97.51, G97.52, H59.111, H59.112, H59.113, H59.119, H59.121, H59.122, H59.123, H59.129, H59.311, H59.312, H59.313, H59.319, H59.321, H59.322, H59.323, H59.329, H95.21, H95.22, H95.41, H95.42, I97.410, I97.411, I97.418, I97.42, I97.610, I97.611, I97.618, I96.620, J95.61, J95.62, J95.830, J95.831, K91.61, K91.62, K91.840, K91.841, L76.01, L76.02, L76.21, L76.22, M96.810, M96.811, M96.830, M96.831, N99.61, N99.62, N99.820, N99.821 |
|  |  |  |

Supplemental Table 2. Factors associated with 80+ age group (reference: 18-49) among patients undergoing nonelective, inpatient cholecystectomy cases for acute cholecystitis. Model C-statistic: 0.89. *Ref: Reference. AOR: Adjusted odds ratio. CI: Confidence Interval.*

| **Parameter** | **AOR [95% CI]** | **p-value** |
| --- | --- | --- |
| Female sex (ref: male) | 0.90 [0.86-0.95] | <0.001 |
| *Race* |  |  |
| White | Ref |  |
| Black | 0.21 [0.19-0.23] | <0.001 |
| Hispanic | 0.66 [0.62-0.71] | <0.001 |
| Asian | 1.46 [1.29-1.65] | <0.001 |
| Other | 0.54 [0.48-0.62] | <0.001 |
| *Income Quartile* |  |  |
| Fourth (highest) | Ref |  |
| Third | 0.79 [0.74-0.84] | <0.001 |
| Second | 0.68 [0.64-0.73] | <0.001 |
| First (lowest) | 0.56 [0.52-0.60] | <0.001 |
| *Comorbidities* |  |  |
| Elixhauser Comorbidity Index | 1.05 [1.03-1.08] | <0.001 |
| Diabetes | 1.08 [1.01-1.17] | 0.03 |
| Hypertension | 7.90 [7.44-8.39] | <0.001 |
| Coronary artery disease | 6.90 [6.24-7.63] | <0.001 |
| Congestive heart failure | 2.69 [2.39-3.03] | <0.001 |
| Chronic lung disease | 0.91 [0.85-0.98] | 0.03 |
| Chronic kidney disease | 0.06 [0.05-0.07] | <0.001 |
| *Concomitant Diagnosis* |  |  |
| Choledocholithiasis | 1.35 [0.92-1.98] | 0.13 |
| Biliary colic | 0.76 [0.54-1.09] | 0.13 |
| Gallstone pancreatitis | 1.25 [1.16-1.34] | <0.001 |
| Cholangitis | 2.88 [2.44-3.39] | <0.001 |
| Tokyo grade III | 3.84 [3.56-4.15] | <0.001 |
| Year of admission (per year) | 1.03 [1.02-1.04] | <0.001 |
| *Treatment Characteristics* |  |  |
| Preoperative ERCP | 2.11 [1.97-2.26] | <0.001 |
| Intraoperative cholangiogram | 1.14 [1.07-1.22] | <0.001 |

Supplemental Table 3. Adjusted outcomes associated with age group among patients undergoing nonelective, inpatient cholecystectomy cases for acute cholecystitis. *AOR: Adjusted odds ratio. CI: Confidence Interval.*

| **Outcome** | **Age Group** | **AOR [95% CI]** | **p-value** |
| --- | --- | --- | --- |
| Major adverse events | 18-49 years | Ref |  |
|  | 50-64 years | 1.48 [1.43-1.54] | <0.001 |
|  | 65-79 years | 1.93 [1.84-2.02] | <0.001 |
|  | 80+ years | 2.51 [2.38-2.65] | <0.001 |
| Conversion to open | 18-49 years | Ref |  |
|  | 50-64 years | 1.90 [1.83-1.97] | <0.001 |
|  | 65-79 years | 2.02 [1.92-2.12] | <0.001 |
|  | 80+ years | 2.03 [1.92-2.16] | <0.001 |
| Repair of bile duct injury | 18-49 years | Ref |  |
|  | 50-64 years | 1.30 [1.08-1.57] | 0.01 |
|  | 65-79 years | 1.59 [1.21-2.08] | 0.001 |
|  | 80+ years | 1.88 [1.39-2.53] | <0.001 |
